# Immunoglobulin A Nephropathy. Recurrence After Renal Transplantation

**DOI:** 10.3389/fimmu.2019.01332

**Published:** 2019-06-19

**Authors:** Gabriella Moroni, Mirco Belingheri, Giulia Frontini, Francesco Tamborini, Piergiorgio Messa

**Affiliations:** ^1^Dialysis, and Renal Transplant Unit, Fondazione IRCCS Ca' Granda Ospedale Maggiore Policlinico, Milan, Italy; ^2^Department of Clinical Science and Community, Università degli Studi di Milano, Milan, Italy

**Keywords:** IgA nephropathy, kidney transplant, proteinuria, humans, glomerulonephritis IgA, prognosis, therapy

## Abstract

IgA nephropathy (IgAN) is the most common primary glomerular disease worldwide. The disease generally runs an indolent course but may lead to ESRD in 20–30% of patients in 20 years or more after diagnosis. Patients with IgA nephropathy are ideal candidates for renal transplant because they are generally relatively young and with few comorbidities. Their graft survival is better or comparable to that of controls at 10 years, though few data are available after 10 years of follow-up. Recurrence of the original disease in the graft is a well-known complication of transplant in IgAN and is a significant cause of deterioration of graft function. Recurrent IgAN rarely manifests clinically before 3 years post transplantation. Recurrence rate is estimated to be around 30% with considerable differences among different series. Despite these factors there is no certain recurrence predictor, young age at renal transplant, rapid progression of the original disease and higher levels of circulating galactose-deficient IgA1 and IgA-IgG immune complexes are all associated with a higher rate of recurrence. Which pathogenetic mechanisms are responsible for the progression of the recurrence to graft function deterioration, and what therapy can prevent or slow down the progression of the disease in the graft, are open questions. The aim of this review is to describe the clinical outcome of renal transplantation in IgA patients with attention to the rate and the predictors of recurrence and to discuss the available therapeutic options for the management of recurrence.

## Introduction

Glomerulonephritis (GN) is one of the most common diseases responsible for ESRD in the renal registries worldwide with an incidence ranging from 10.5 to 38.2% and a prevalence from 17.6 to 53.5% (1). In Australia, in the period between 2010 and 2013, IgAN was responsible for 16% of all incidents ESRD (2). Kidney transplantation is the best therapeutic option for IgAN patients. They have greater access to kidney transplant compared to patients with other kidney diseases because they are generally young and with few comorbidities. In the United States, between 2007 and 2011, the proportion of patients who received a kidney transplant within 1 year was 30.2% for IgAN compared to 2.4% for those with diabetes (3).

IgAN frequently recurs in the graft. Owing to the progressive improvement of graft survival resulting from reduced incidence of acute rejection, the number of patients potentially exposed to recurrence has increased. The incidence of IgAN recurrence is not clearly estimated; it is reported to range from 9 to 53% (4, 5), but the incidence increases if we consider only patients submitted to graft biopsy. Several reasons can account for this large discrepancy. The variable indication to graft biopsy in the different centers is one of the reasons. The rate of IgAN recurrence is higher in studies in which protocol biopsies are performed (5) in comparison to studies that reported graft biopsies done for clinical reasons only (6, 7) and in Registry analysis (4). As a matter of fact, recurrences of IgAN can be “histological only” when diagnosed on protocol biopsies in asymptomatic patients; or “clinical,” when associated with urinary abnormalities and/or graft dysfunction. The duration of post-transplant follow-up is another important factor for the development of recurrence of IgAN because the rate of recurrence increases as the time from transplant lengthens (5). It is unclear if recurrence is (8–10) or is not (11, 12) more frequent in transplants using related donors but this uncertainty does not preclude consideration of living donation in IgAN.

Recurrent glomerulonephritis is a significant cause of graft loss in transplant patients. Largest registry studies of Australia and New Zealand reported that IgAN recurrence was a significant cause of graft loss, third only to chronic allograft nephropathy and death with graft function. At 10 years the rate of graft loss for IgAN was similar to that of other forms of glomerulonephritis and to those of other causes of ESRD (13, 14). Outcomes after 10 years have been insufficiently studied in registry studies to draw firm conclusions, although results of a recent European Registry analysis (15) and some cohort studies suggested increasing graft loss after 10 years (16, 17).

## IgA Nephropathy in the Native Kidney

Among primary chronic glomerular diseases, IgA nephropathy (IgAN) is the most prevalent worldwide (18). The clinical presentation is highly heterogeneous ranging from macroscopic hematuria with or without acute renal dysfunction during an infective episode and microscopic hematuria, isolated, or associated with proteinuria and hypertension. The diagnosis of certainty requires a renal biopsy, which shows the presence of dominant or codominant diffuse mesangial deposition of IgA which, in most cases, are aberrantly glycosylated. The mesangial IgA is exclusively of the IgA1 subclass and is deficient in galactose (19).

The pathogenesis of the disease is not completely clarified. Recent genome-wide association studies have identified multiple susceptibility loci for IgAN implicating independent defects in adaptive and innate immunity, and alternative complement pathways that potentially influence the different pathogenetic steps of the disease (20). By combining genetic and biochemical data, at least four processes seem to contribute to the development of the disease. Based on the available knowledge, there is in IgAN a primary, inherited defect that leads to preferential production of IgA1 with galactose-deficient O-glycans in the hinge-region (21). In the presence of inheritance of permissive MHC haplotypes, IgA1 deficient in galactose elicits the production of antiglycan autoantibodies, that lead to the formation and subsequent glomerular deposition of immune complexes. In particular IgA based activation of alternative complement pathway play a critical role in the IgAN pathogenesis and C3 is frequently involved in the formation of circulating immune deposits inducing mesangial stress, podocyte damages (22), and progressive deterioration of kidney function (23). On this basis IgAN is classified as an autoimmune disease (24). Recent attempts to characterize mediators of this response have focused on facilitators of B-cell differentiation and proliferation (25). The B cell activation factor of TNF family (BAFF) and a proliferation-inducing ligand (APRIL) has been indicated as modulating factors of B cell in the IgA pathogenesis by reducing both IgA serum levels and IgA glomerular deposition. These molecules may represent intriguing therapeutic targets for new IgAN treatments (26).

The disease generally runs an indolent course with a 10-year renal survival rate of about 90% among adults (27) and children (28) with normal renal function at diagnosis. However, 20–30% of patients develop end stage renal disease (ESRD) after 20 years or more follow-up (29), and need chronic dialysis or renal transplantation.

Proteinuria of at least 1 g/day, decreased estimated glomerular filtration rate (eGFR) at diagnosis and uncontrolled arterial hypertension are the clinical risk factors for the progression to ESRD (30). The Oxford classification of histological features of IgAN in the native kidney, that is based on the semiquantitative score of four histological features (mesangial hypercellularity, segmental glomerulosclerosis, endocapillary hypercellularity, and tubular atrophy/interstitial fibrosis), was found to be associated with increased risk of eGFR loss independent of clinical variables (31).

## Histological Features of IgAN Recurrence in Kidney Transplant

Biopsy is mandatory not only to diagnose the disease in the native kidney, but also to identify and characterize graft recurrence of IgAN in the kidney graft. For this reason, the biopsy policies of the different centers and the techniques of histological evaluation have an important role in the definition of presence, frequency, and type of recurrence. Recurrence can be histological only, characterized by mesangial IgA with or without mesangial proliferation in the absence or presence of mild clinical manifestations. Ortiz et al. (32) evaluated the incidence of IgAN recurrence through protocol biopsies in 65 patients and found that the histological recurrence of IgAN can be recognized in almost one-third of patients within 2 years from transplantation. These results confirm those of a previous study with protocol biopsies that reported a 53% recurrence rate in 32 IgAN transplanted patients. Most of these patients did not have clinical signs of recurrence (5). The clinical value and the outcome of silent recurrent IgA deposits is not completely clarified. In some cases the disease progressed to overt clinical IgAN recurrence, (33) in others, IgA deposits disappeared in subsequent biopsies (34). The possibility of disappearance of IgA deposits from the kidney was also demonstrated in repeated renal biopsies of native IgAN (35). The demonstration that some apparently normal donors (living or deceased) may have latent IgA deposits in the kidney, raises the possibility that some histological recurrence can have such origin (36). Actually, when allografts with latent mesangial IgA deposits were transplanted in non IgA patients, the IgA was rapidly cleared by the recipients as demonstrated in follow-up biopsies (37). The transformation of the histological recurrence in clinical recurrence could be mediated by the activation of accessory factors such as activation of complement and cytokines production (38). However, based on the few available data about the clinical value of “histological” recurrence and the lack of an effective therapy, protocol biopsies in renal transplant patients with IgAN are not recommended unless steroid withdrawal is considered. The histological findings recorded at graft biopsy performed for clinical reasons, are not different from those of the original disease. In graft biopsy the diagnosis can be complicated by the simultaneous presence of other glomerular or vascular lesions secondary to acute or chronic graft rejections. In these cases, not only light microscopy and immunofluorescence, but also electron microscopy is needed to clearly define the possible association of two different diseases. In addition to mesangial IgA immunodeposits at immunofluorescence, mesangial proliferative glomerulonephritis is the typical histological pattern at light microscopy in IgAN recurrence with, in a minority of cases, features of glomerular cellular crescents. In a cohort of 71 IgAN transplanted patients, cellular or fibrocellular crescents were demonstrated in 14.1% of graft biopsies and were associated with a higher degree of interstitial inflammation and with a significant worse graft survival (39).

Some studies suggest that, the Oxford classification of histological features of IgAN in the native kidney, (31) applied to biopsies of recurrent IgAN has prognostic value for graft failure (40, 41). Based on these results the evaluation of the Oxford classification score, should be performed in all cases of IgAN recurrence.

## Clinical Manifestations of IgAN Recurrence in Kidney Transplant

In the majority of studies the IgAN recurrence is diagnosed in graft biopsies performed for clinical reasons. The clinical presentation and the outcome of recurrences is extremely variable. Hematuria, the hallmark of IgAN in the native kidney, is not a reliable manifestation in recurrence as it was not present in 64% of cases diagnosed on protocol biopsies (32). Even in biopsies performed for clinical reasons hematuria is not the rule. In our cohort of 190 IgAN transplanted patients, the 43 recurrences presented with isolated microscopic haematuria in 5, isolated proteinuria in 8, microscopic haematuria and proteinuria in 12, isolated increase in plasma creatinine in 4, increase in plasma creatinine associated with haematuria or haematuria and proteinuria in the other 13 cases. In the four patients with an isolated increase in serum creatinine proteinuria and haematuria developed later, instead in seven patients all the urinary abnormalities regressed during the follow-up (17). Altogether in about one quarter of our patients, microscopic haematuria, the hallmark of IgAN in the native kidney, was not present at diagnosis of recurrences. This underlines that graft biopsy is essential to ensure the correct diagnosis.

## Incidence of IgAN Recurrence in Kidney Transplant

The reported incidence of IgAN recurrence in biopsies performed for clinical reasons is around 30% but with a range of 9–53% among the different series if we consider protocol and clinical biopsies (Table 1). This large discrepancy can be attributed to several reasons in addition to the above reported different biopsy policies and techniques of histological evaluation in the different centers. The different racial and geographical distributions of IgAN may influence the rate of recurrence. The presence of IgA deposits in donated kidneys, frequently observed in Japanese series (36) was hypothesized to increase the probability of recurrence (47). The rate of recurrence can be underestimated if all patients who receive a renal transplant do not have a histological diagnosis of the original disease, or if the follow-up after transplant is too short. Recurrence rate reported in the ANZDATA registry for IgAN at 5 and at 10 years were, respectively 5.4% (4.3–6.5) and 10.8% (9.2–12.5%) (4). These data suggest that, recurrence of IgAN is apparently a time dependent event; the longer the follow-up, the higher the probability of recurrence. The incidence of IgAN recurrence can be underestimated if all patients with deterioration of renal function or with proteinuria are not submitted to graft biopsy. In the absence of graft biopsy, it is possible that some graft losses classified as chronic allograft nephropathy could be cases of unrecognized recurrence of IgAN (13).

**Table 1:** Main studies on IgA nephropathy recurrence.

**Year of publication**	**Authors**	**Study design**	**Number of patients**	**IgAN recurrence rate (%)**	**Time to recurrence (years)**	**Graft losses due to recurrence (%)**	**Follow–up (years)**	**Protocol or clinical biopsies**
1998	Matsugami et al. (42)	Retrospective	49	24.5	–	–	–	Protocol
2007	Mousson et al. (43)	Retrospective	42	52.4	–	4.7	8.9	Protocol
2012	Ortiz F et al. (32)	Retrospective	65	32.3	6.9 months	3	4.67	Protocol
1994	Odum et al. (5)	Prospective–retrospective	46	53 36.9	3.8	17.2	3–183 months	Protocol/ Clinical
1996	Kessler et al. (33)	Retrospective	84	15.5	3.2	4.8	5.7	Clinical
1997	Frohnert et al. (34)	Retrospective	53	26.4	–	18.8	–	Clinical
1997	Ohmacht et al. (44)	Retrospective	61	22.9	5.4	16.4	–	Clinical
1998	Bumgardner et al. (44)	Retrospective	61	29.5	2.6	9.8	5.1	Clinical
1999	Freese et al. (6)	Retrospective	104	12.5	6	5.8	5	Clinical
2000	Stack et al. (45)	Retrospective	44	11.4	–	–	–	Clinical
2001	Kim et al. (12)	Retrospective	90	21.1	–	2.2	–	Clinical
2001	Wang et al. (9)	Retrospective	48	29.2	4.3	8.3	4.3	Clinical
2001	Ponticelli C et al. (11)	Retrospective	106	34.9	4.3	3.8	5.9	10 Protocol 47 Clinical
2001	Andresdottir et al. (46)	Retrospective	79	21.5	13–145 months	1.3	5.6	Clinical
2003	Choy et al. (16)	Retrospective	75	18.7	5.6	4	–	Clinical
2005	Moriyama T et al. (47)	Retrospective	49	26.5	3.25	10	–	Clinical
2005	Chandrakantan et al. (7)	Retrospective	152	13.1	3.6	7.8	–	Clinical
2005	Soler et al. (48)	Retrospective	37	19.4	4.1	8.1	–	Clinical
2009	Kennoki et al. (49)	Retrospective	405	16	–	–	1	Protocol
2009	Han et al. (10)	Retrospective	221	19.9	–	7.7	5.9	Clinical
2012	Kamal et al. (50)	Retrospective	142	17.6	–	7	6.6	Clinical
2013	Moroni et al. (51)	Retrospective	190	22.1	3.7	6.3	9.4	Clinical
2014	Von Visger et al. (52)	Retrospective	124	22	–	–	6.8	Clinical
2017	Berthoux et al. (53)	Retrospective	96	35.4	5.8	12.3	12.4	Clinical
2017	Avasareet al. (54)	Retrospective	62	22.5	2.75	–	–	Clinical
2018	Garnier et al. (55)	Retrospective	67	20.8	3.13	–	5.9	Clinical
2018	Jiang et al. (4)	Retrospective	2,393	9.9	4.63		10	Clinical

The rate of graft loss due to recurrence is reported to range from 1.3 (46) to 17% (5). However, the attribution of graft loss to the recurrence is not easy due to the possible presence of concomitant lesions due to acute or chronic graft rejections or to calcineurin inhibitors toxicity. This is particularly true if graft biopsy is not available close to the graft function deterioration (56).

## Predictors of IgAN Recurrence in Kidney Transplant

Analysis of risk factors for IgAN recurrence have not provided consistent results. Young age at renal transplantation, male gender, and rapidly progressive course of the original disease before transplantation were associated with a higher risk of recurrence in some studies (10, 11, 13, 57). Avesare et al. (54) reevaluated the clinical and biopsy features of native kidney in 62 IgAN patients who received a transplant at Columbia University Medical Center from 2001 to 2012. They found that younger age at onset of IgAN and greater burden of crescents on native biopsy predicted recurrence after transplant.

Garnier et al. (55) found that serum IgA levels 6 months post-transplant was a risk factor of IgAN recurrence, independently of age, and serum creatinine. This was particularly true in patients who had serum IgA levels above 222.5 mg/dL at month 6 post-transplant.

Berthoux et al. (53) tested serum autoantibodies specific for galactose deficient-IgA1 at time of transplant or at time of native-kidney diagnosis in 96 first renal transplant recipients with IgAN. This study demonstrated that normalized serum levels of galactose deficient -IgA1–specific IgG autoantibody was an independent risk factor for recurrence of the disease in the allograft. This was particularly true for the earlier recurrences: 75% of events occurring before 8.79 years after transplantation (53).

Another unsolved question is whether living related donor kidneys have a higher risk of recurrence than kidneys from non-related donors. Some studies have failed to detect a significant difference (7, 11, 12, 34, 58) while others noted more frequent recurrences in grafts from living related donors (6, 9, 10). A review of the Australia-New Zealand registry data reported that IgAN recurrence was significantly more frequent in zero HLA mismatched living donor grafts (59). However, these results should be interpreted with caution because several details are lacking in retrospective reviews of registry data.

The risk of recurrence in the second graft in glomerulonephritis patients who lost the first graft for recurrence was investigated in 7,236 patients of the ANZDATA transplant registry. No increased risk of further recurrence in a subsequent graft was demonstrated for IgAN patients (4). This result is in contrast with previous small reports which suggested an increased risk of recurrences in patients with IgAN who lost the first graft for recurrence (6, 44, 60)

Current immunosuppressive agents used in transplantation do not seem to reduce IgAN recurrence in the allograft. Retrospective data from a single center suggests that induction therapy with antithymocyte globulin prevents the development of IgAN recurrence (57). Von Visger et al reported a significantly higher rate of IgA recurrence in patients maintained on steroid-free immunosuppression in comparison to those who received steroid-based immunosuppression (52).

## Patient and Renal Survival of Kidney Transplant in IgAN Patients

Outcome of patients and graft survival in IgAN can be obtained from Registry analysis and from single centers observational studies. In comparison to single center studies, Registry analysis have the advantage of reporting data on a high number of patients but, some outcomes, such as causes of allograft failure, and incidence of recurrence are not accurately measured or are underestimated due to the different policies of the included centers. Single center retrospective studies provide more detailed information on different aspects of the disease in IgA transplanted patients but have low power in predicting the outcome.

The survival of transplanted patients with IgAN seems to be comparable or slightly better with that of recipients with other renal diseases both in Registry analysis and in single center studies. Among 32,131 patients with glomerulonephritis transplanted in the United States between 1996 and 2011, patients with IgAN had the lowest mortality rates (10% mortality at 10 years) in comparison to other glomerulonephritis forms and a slightly better survival than that of transplanted patients with adult polycystic kidney disease (APKD) (61). UK Renal Registry data, that analyzed patient survival in 2,975 incident patients with primary glomerulonephritis in comparison to 1,775 transplanted APKD patients who received a renal transplant between 1997 and 2009, reported unadjusted 10-year patient survival of 80.7% for APKD and 85.6% for IgAN (62).

Instead, the results of graft survival in patients with IgAN have been differently estimated across different countries and populations. The UNOS/OPTN database, in patients transplanted between 1999 and 2008, reported, in patients stratified for donor type (living vs. deceased) that at multivariable regression analysis the adjusted HR for death-censored allograft survival was not significantly different between IgAN and non-IgAN patients (63). Data from the European ERA-EDTA registry reported that the risk of death-adjusted graft failure for IgAN patients was not different from that of APKD until 10 years after transplant, thereafter the risk of graft loss was greater in IgAN (15). In addition, this study demonstrated that living related transplants in IgAN patients had lower risk of death-adjusted graft failure compared with deceased related transplants. The same lower death-adjusted graft survival of IgAN patients in comparison to APKD was reported by a largest study of transplanted patients in the United States (61).

The results concerning graft survival of IgAN patients reported by the single center studies are equally contrasting and depend partly on the length of the observation.

Some investigators reported a more favorable graft survival in IgAN patients than in other transplant recipients (64, 65), but other authors found similar results in an IgAN group than in non-IgAN transplant patients (48, 50, 58, 66). In particular, during the first 5 years after transplantation allograft survival for primary IgAN patients seems to be better than that of recipients with other primary renal diseases (12, 46, 64, 66, 67). Based on these short-term results, the recurrence of IgAN was considered to have little or no impact on graft function or on patient outcome (5, 68). At 10 years, graft survival of IgAN patients became comparable to that of recipients with other renal diseases (11, 12).

Choy et al. (16) found that the graft survival of up to 10 years was worse in IgAN patients than in controls, the two survival curves crossover at around 12 years. Our results are in agreement with actuarial death censored graft survival rates at 15 years of 62.6% in IgAN patients significantly worse than the 72.4% in the control group. In our cohort, the difference between the two survival curves become significant after the 15th year. When we separately evaluated the outcome of patients with and without recurrence we found that death censored graft survival at 15 years was 51.2% in the recurrent patients and significantly better (68.3%) in the non-recurrent recipients. Finally, graft survival in non-recurrent patients was not different from that of controls (17). The worst graft survival in the long-term of recurrent in comparison to non-recurrent IgAN patients was confirmed both in the recent report of the ANZDATA Registry (4) and by a retrospective study in 221 Korean recipients. In this study around 50% of patients received a graft from living related donors. Ten-years graft survival was 61% in recurrent IgAN group vs. 85.1% in non-recurrent patients (10).

It is well-known, that recurrence of glomerulonephritis is the third most frequent cause of allograft loss at 10 years, after chronic rejection and death with a functioning graft (13, 69). Based on these recent results, it seems that also for IgAN, the graft recurrence represents a substantial risk factor for graft failure in the very long-term as hypothesized by Ponticelli and Glassock (70). The introduction of new and more effective immunosuppressive agents has reduced graft loss by decreasing the incidence of acute rejection and of interstitial fibrosis and tubular atrophy. In this scenario, the recurrence of glomerulonephritis became progressively more frequent and can be a significant cause of graft loss in clinical practice. Mulay et al. reported that, after adjusting for important covariates, the use of cyclosporine, tacrolimus, azathioprine, mycophenolate mofetil, sirolimus, or prednisone does not prevent graft failure due to recurrent glomerulonephritis, including IgAN. However, any change in immunosuppression during follow-up was independently associated with graft loss due to recurrence (71).

Probably, old and short-term studies failed to detect a clinical impact of recurrent disease because, it is rare that the function of a graft with IgAN recurrence deteriorates during the first years after transplantation (67).

## Treatment of Graft Recurrences of IgAN Patients

No universally accepted guidelines for the treatment of recurrence of IgAN in renal transplant are currently available. The goal of the therapy should be to prevent recurrence of IgAN.

Clayton et al. (14) suggested that steroid withdrawal may increase graft loss risk because of recurrence of IgAN. These results were confirmed in two subsequent studies (72, 73). In a retrospective analysis of the UNOS/OPTN database that included 9,690 IgAN adults who received their first kidney transplant between 2000 and 2014, early steroid-withdrawal was associated with an increased risk of recurrence at multivariate analysis (72).

At present treatment should aim to reduce proteinuria, to optimize blood pressure and to reduce inflammation as suggested by the KDIGO Transplant guidelines (74). The institution of good supportive therapy in particular the use of angiotensin converting enzyme inhibitors or angiotensin II receptor blocker in patients with proteinuria higher than 0.5 g/day is recommended based on good results reported in some studies (75, 76). Careful blood pressure control, particularly in patients with proteinuria > than 1 g/day, seems to be important to preserve graft function. However, it is important to consider that the use of these drugs in renal transplant may cause significant decreases in glomerular filtration rate and hematocrit (77). Studies from Japan report favorable outcomes after tonsillectomy in patients with recurrent IgAN (49, 78, 79) but these results need to be confirmed in different ethnicities. In the few cases of recurrent IgAN with rapid progressive renal insufficiency and crescents at biopsy, a rescue treatment with high-dose corticosteroids with cyclophosphamide, and or plasmapheresis may be attempted, although the results are usually poor. A promising therapeutic option is represented by drugs which target directly the intestinal mucosa, such as budesonide. Fellström et al. reported a reduction of proteinuria and a stabilization of renal function after budesonide administration in IgAN patients, suggesting a possible role of this drug also in patients with IgAN recurrence after kidney transplant (80).

## Conclusions

Despite the high rate of recurrence and its potential negative impact on graft survival, renal transplant is the best choice for patients with ESRD secondary to IgAN. In our experience, recurrence of IgAN significantly reduced from 1981 to 2010 and in particular, recurrences occurred less frequently in transplants performed during the decade 2000–2010 (17). These results and the apparent reduction in graft loss from recurrent IgAN reported by Mulay et al. (71) and by Clayton et al. (14) in recent years may portend a progressive improvement of prognosis of renal transplant in IgAN patients in the near future. Better understanding of pathogenesis of the disease may enable more “specific” therapy. A large prospective multicenter randomized controlled trial with a long follow-up to evaluate interventions for recurrent IgA would be welcome and of importance in helping clinicians and patients.

## Author Contributions

GM, MB, GF, FT, and PM conceived the article contents, prepared the manuscript, and endorsed the final draft submitted.

### Conflict of Interest Statement

The authors declare that the research was conducted in the absence of any commercial or financial relationships that could be construed as a potential conflict of interest.
